# Polo-like Kinase 4: A Multifaceted Marker Linking Tumor Aggressiveness and Unfavorable Prognosis, and Insights into Therapeutic Strategies

**DOI:** 10.3390/cancers15184663

**Published:** 2023-09-21

**Authors:** Youngtaek Kim, Joon Yeon Hwang, Dong Kwon Kim, Kwangmin Na, Seul Lee, Sujeong Baek, Seong-san Kang, Seung Min Yang, Mi Hyun Kim, Heekyung Han, Chai Young Lee, Yu Jin Han, Min Hee Hong, Jii Bum Lee, Sun Min Lim, Byoung Chul Cho, Youngjoon Park, Kyoung-Ho Pyo

**Affiliations:** 1 Department of Research Support, Yonsei Biomedical Research Institute, Yonsei University College of Medicine, Seoul 03186, Republic of Korea; kyt1997@yuhs.ac (Y.K.); jyhwang4@yuhs.ac (J.Y.H.); juneluna@yuhs.ac (K.N.); sbaek@yuhs.ac (S.B.); ssmm51@yuhs.ac (S.M.Y.); mijareng@yuhs.ac (M.H.K.); winsca@yuhs.ac (H.H.); cyl3165@yuhs.ac (C.Y.L.); ginnahan@yuhs.ac (Y.J.H.); 2 Severance Biomedical Science Institutse, Yonsei University College of Medicine, Seoul 03186, Republic of Korea; vious666@yuhs.ac (D.K.K.); seullee@yuhs.ac (S.L.); 3 Brain Korea 21 PLUS Project for Medical Science, Yonsei University College of Medicine, Seoul 03186, Republic of Korea; 4 JEUK Institute for Cancer Research, JEUK Co., Ltd., Gumi 39418, Republic of Korea; skang@yuhs.ac; 5 Division of Medical Oncology, Yonsei Cancer Center, Yonsei University College of Medicine, Seoul 03186, Republic of Korea; minhee_hong@yuhs.ac (M.H.H.); joy88lee@yuhs.ac (J.B.L.); limlove2008@yuhs.ac (S.M.L.); cbc1971@yuhs.ac (B.C.C.); 6 Yonsei New Il Han Institute for Integrative Lung Cancer Research, College of Medicine, Yonsei University, Seoul 03186, Republic of Korea; 7 Department of Medical Science, College of Medicine, Yonsei University, Seoul 03722, Republic of Korea

**Keywords:** lung adenocarcinoma, PLK4, *TP53*, biomarkers, drug resistance

## Abstract

**Simple Summary:**

Polo-like kinase 4 (PLK4) is associated with tumorigenesis and prognosis in various types of cancer. Prognostic analysis of *PLK4* expression and data analysis of its association with the somatic mutation and drug resistance through clustering were performed using the Cancer Genome Atlas-lung adenocarcinoma (TCGA-LUAD) dataset. According to these results, *PLK4* expression is associated with poor prognosis, *TP53* mutations, and drug resistance in patients with LUAD.

**Abstract:**

(1) Background: This study investigated whether polo-like kinase 4 (PLK4) is a suitable therapeutic target or biomarker for lung adenocarcinoma (LUAD). (2) Methods: We acquired LUAD data from The Cancer Genome Atlas (TCGA) database through the UCSC Xena data portal. Gene expression, clinical, survival, and mutation data from multiple samples were analyzed. Gene enrichment analysis, unsupervised clustering of *PLK4*-related pathways, and differential gene expression analyses were performed. Additionally, correlations, *t*-tests, survival analyses, and statistical analyses were performed. (3) Results: *PLK4* expression was higher in LUAD tissues than in normal tissues and was associated with poor prognosis for both overall and progression-free survival in LUAD. *PLK4* was highly correlated with cell-proliferation-related pathways using Gene Ontology (GO) biological process terms. *PLK4* expression and pathways that were highly correlated with *PLK4* expression levels were upregulated in patients with LUAD with the *TP53* mutation. (4) Conclusions: *PLK4* expression affects the survival of patients with LUAD and is a potential therapeutic target for LUAD with *TP53* mutations.

## 1. Introduction

Lung cancer is one of the most common cancers worldwide, causing approximately 130,000 deaths annually [1,2,3]. Histologically, lung cancer can be divided into non-small-cell lung cancer (NSCLC) and small-cell lung cancer (SCLC) [4]. NSCLC accounts for 80% of all lung cancers and includes the following major types: adenocarcinoma (32–40%), squamous cell (25–30%), and large cell (8–16%) [5]. Lung adenocarcinoma (LUAD), the major subtype of NSCLC, originates from epithelial cells, the major cell type in lung tissue, and usually occurs in the periungual area [6]. The etiology of LUAD involves multiple factors and genetic variations [7]. Smoking is one of the major causes of LUAD; however, it can also occur in non-smokers and may result from exposure to harmful environmental substances and genetic predisposition [8,9,10]. Despite significant advances in cancer treatment, LUAD still poses significant challenges due to factors such as drug resistance [11], tumor heterogeneity [12], metastasis [13], and immune evasion [14]. Recent studies have highlighted the pivotal role of polo-like kinase 4 (PLK4) in the LUAD therapeutic target to address these challenges [15,16].

PLK4, a member of the polo-like kinase family, is characterized by a highly conserved catalytic domain with serine/threonine kinase activity [17,18]. Structurally, PLK4 consists of an N-terminal kinase domain, a central linker region, and a C-terminal non-catalytic domain [19]. This unique architecture allows PLK4 to interact with various binding partners and regulatory factors, thereby enabling precise spatiotemporal control during the cell cycle [20]. Centrioles are small, cylindrical organelles that form the core of centrosomes and are essential for organizing mitotic spindles during cell division [21]. PLK4 is the key regulator of centriole duplication, initiating the assembly of daughter centrioles adjacent to pre-existing mother centrioles [20]. Dysregulation of PLK4 activity leads to abnormal centriole numbers, resulting in the formation of supernumerary centrosomes, genomic instability, and cancer development [22]. Aberrant *PLK4* expression is frequently observed in cancer and contributes to tumorigenesis and unfavorable patient prognosis [18]. However, investigations into the correlation between *PLK4* expression and tumor growth and prognosis in LUAD are limited [16]. In particular, studies have been conducted on the biological function of PLK4 in cancer apoptosis and its association with cancer prognosis [23,24,25], but still limited in LUAD. Although there have been previous studies on the expression of PLK4 in LUAD [15,16], additional evidence was needed as to whether PLK4 is suitable as a therapeutic target for LUAD. Thus, in this study, we analyzed the association between *PLK4* and LUAD using the Cancer Genome Atlas (TCGA) database.

## 2. Materials and Methods

### 2.1. Public Omics Database Acquisition for Analysis

For bulk RNA sequencing (RNA-seq) data acquisition, we retrieved LUAD data from TCGA database using the UCSC Xena data portal (https://xenabrowser.net/; accessed on 30 July 2023). The dataset encompasses gene expression information of 576 samples (version 2017-10-13) supplemented by clinical data of 706 samples (version 2019-12-06), survival data of 641 samples (version 2018-09-13), and whole-exome sequencing data detailing non-silent somatic mutations across 513 samples (version 2016-12-29). In the mutation data, ‘0’ indicates the wild type, whereas ‘1’ indicates the presence of a mutation. The expression data underwent level 3 processing, involving log2 transformation (log2(x + 1)) following RSEM normalization. The final analysis included 576 samples of all four datasets. Whole-exome sequencing (WES) with non-silent mutations was generated from Multi-Center Mutation Calling in Multiple Cancers Project [26]. Cancer Cell Line Encyclopedia (CCLE) data were downloaded as ‘Expression public 23Q2 data’ from the DepMap database (https://depmap.org/portal/; accessed on 30 July 2023) (version DepMap Public 23Q2).

### 2.2. Gene Enrichment Analysis

Gene enrichment analysis was conducted using ClueGO (version 2.5.8) module in Cytoscape (version 3.9.0). GO (Gene Ontology) biological process was employed for the analysis, and the minimum and maximum intervals of the GO tree were set to 3 and 4, respectively. A minimum of five genes were required, and Bonferroni correction was applied for multiple comparisons.

### 2.3. Single Sample Gene Set Enrichment Analysis

We performed gene enrichment analysis using a dataset consisting of 514 primary tumor samples, each containing gene expression and clinical data. The GO terms obtained from the previous analysis of GO biological processes were analyzed based on the gene set corresponding to each term. We used gene sets associated with each GO term. The entire analysis was implemented using Python (version 3.8), with the utilization of the ‘gseapy’ module (version 1.0.5). To assess the enrichment of these gene sets, we employed the ‘ssgsea’ function in the aforementioned module.

### 2.4. Unsupervised Clustering

To identify the expression patterns of *PLK4*-related pathways, we conducted agglomerative clustering using 20 features. Euclidean distance was calculated using the Ward method after standard scaling of the scores of the 20 *PLK4*-related pathways. We used the sklearn package (version 0.0.1) in Python (version 3.8).

### 2.5. Differentially Expressed Gene (DEG) Analysis and Visualization

We conducted DEG analysis between clusters 1 and 2, with a significance threshold of <0.0001 and log2 fold change >3. The drug–gene interaction database (https://www.dgidb.org/; accessed on 30 July 2023) was used to identify potential druggability. Network analysis and visualization were performed using Cytoscape (version 3.9.0).

### 2.6. Statistical Analysis

A Pearson’s correlation analysis was conducted. The threshold for the correlation coefficient was above 0.8, and the significance level was set at *p* < 0.05. All t-tests were performed using a type-1 error threshold of 0.05. Survival analyses, including overall survival (OS) and progression-free survival (PFS), were conducted using the log-rank test and Cox regression analysis, with a significance level of 0.05. All statistical analyses were performed using R software (version 4.1.2).

## 3. Results

### 3.1. RNA-seq Analysis Workflow

**Figure 1:**
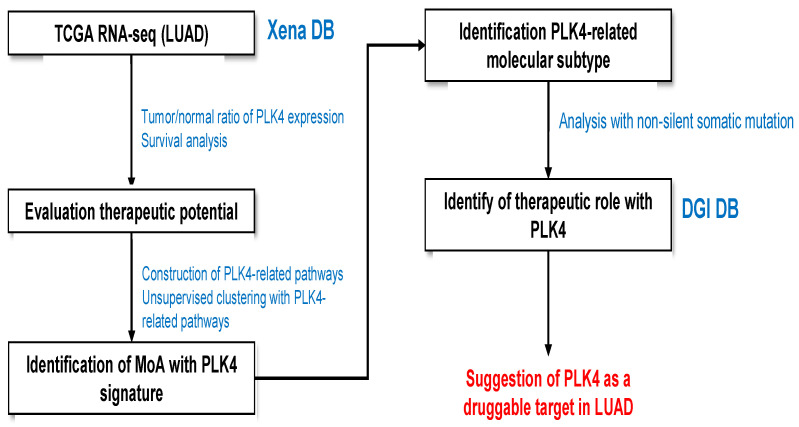
RNA-seq analysis workflow for evaluation of LUAD patient samples.

The workflow of this study is to perform RNA-seq analysis with PLK4 focus using publicly available databases (Figure 1).

### 3.2. Upregulation of PLK4 in LUAD and Association with Poor Prognosis

**Figure 2:**
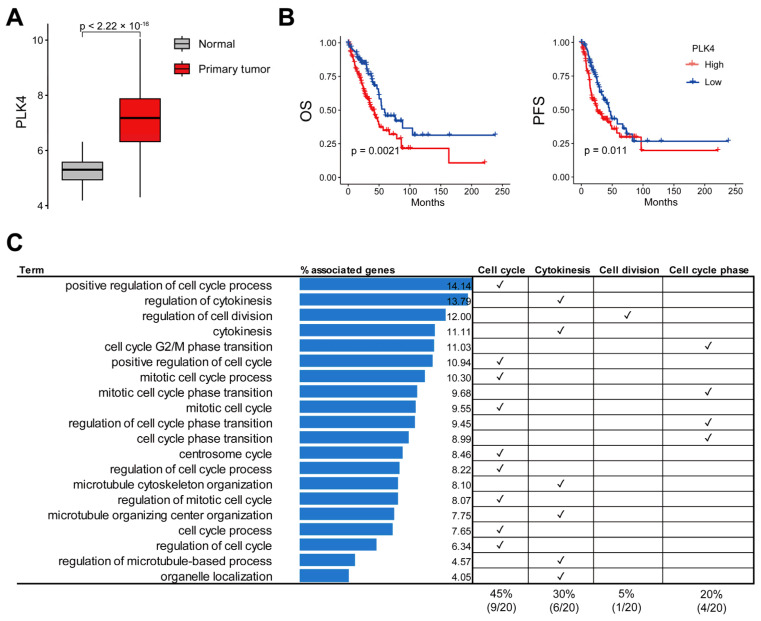
Analysis of *PLK4* gene expression and survival in LUAD based on TCGA database, and identification of genes and signaling pathways with high correlation to *PLK4*. In the expression comparison analysis, 576 samples, with both gene expression data and clinical data, were used; of these, 59 were normal and 514 were primary tumor samples (excluded samples: 2 recurrent tumor samples and 1 FFPE scrolls sample). (**A**) Comparison of *PLK4* gene expression between normal and LUAD primary tumor samples revealed significantly higher expression levels in LUAD primary tumors. (**B**) Survival comparison between the bottom 25% (*n* = 127 samples) and the top 25% (*n* = 126 samples) of *PLK4*-expressing samples in LUAD primary tumor samples showed a significant OS and PFS benefit for the bottom 25% with low *PLK4* expression. (**C**) In the LUAD primary tumor sample, 87 genes with coefficient values above 0.8 and *p*-values below 0.05 that correlated with *PLK4* were identified. Subsequently, a GO biological process analysis was conducted employing these 87 genes, leading to the extraction of relevant terms.

Analysis of the RNA-seq and WES results for *PLK4* in LUAD is shown in Figure 1. To evaluate the role of PLK4 in LUAD, we compared the *PLK4* expression between tumor and normal samples. *PLK4* expression was significantly higher in primary tumors than in normal tissues (*p*-value: 2.22 × 10^−16^) (Figure 2A). 

In total, 576 samples, with both gene expression and clinical data, were used in the expression comparison analysis; of these, 59 were normal samples and 514 were primary tumor samples (2 recurrent tumor samples and 1 formalin-fixed paraffin-embedded (FFPE) scroll sample were excluded). In addition, we divided the two groups into the top 25% and bottom 25% according to the level of *PLK4* expression, and the top 25% was significantly associated with poor prognosis in both OS and PFS compared with the bottom 25% (log-rank test *p*-value < 0.05) (Figure 2B). In addition, it exhibited a risk effect on both OS (hazard ratio: 1.228, confidence interval: 1.077–1.4, and *p*-value: 0.002) and PFS (hazard ratio: 1.193, confidence interval: 1.054–1.35, and *p*-value: 0.005). The samples used for survival analysis included 514 primary tumor samples with *PLK4* gene expression and clinical data and 505 samples with survival data. Among the 505 samples, 126 samples in the top 25% of *PLK4* expression and 127 samples of the bottom 25% were analyzed. To identify PLK4-related pathways, we conducted Pearson’s correlation analysis of *PLK4* expression and whole-gene expression. A total of 87 genes were significantly correlated with *PLK4* expression in LUAD (correlation coefficient > 0.8) (Supplementary Table S1) and were enriched in cell-proliferation-related terms such as cell cycle, centrosome cycle, and regulation of cytokinesis (Figure 2C and Supplementary Table S2).

### 3.3. PLK4-Related Pathways Were Associated with TP53 Mutations

**Figure 3:**
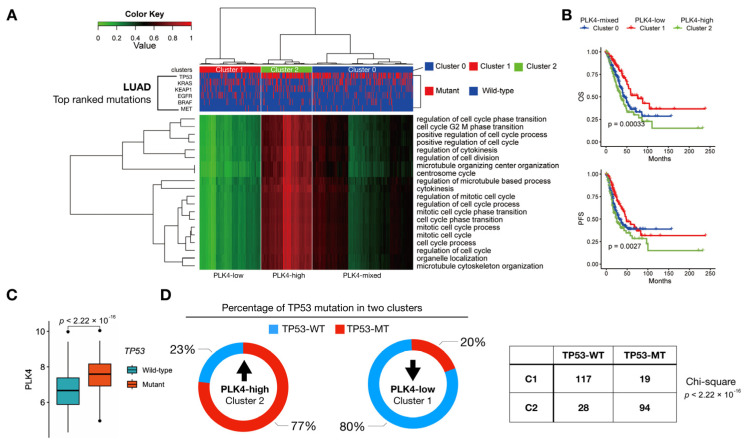
TCGA-database-based single sample gene set enrichment analysis (ssGSEA) results. (**A**) List of signaling pathways related to *PLK4* was derived through correlation analysis using LUAD primary tumor samples. Each term was then scored using ssGSEA with the gene expression information of the sample, and the clusters were divided into three parts using Agglomerative Clustering analysis. The clusters were numbered as 0 (*n* = 240), 1 (*n* = 146), and 2 (*n* = 122). Heatmaps were plotted comparing the ssGSEA information with the cluster information and the top six somatic mutation genes of LUAD. (**B**) Results of survival analysis for the three clusters. (**C**) Comparison of *PLK4* gene expression differences with and without *TP53* mutation. Total number of samples is 505, of which 252 are wildtype and 256 are mutated. (**D**) Proportion of *TP53* mutants in each cluster is shown in a pie chart.

To evaluate the association of *PLK4*-related pathways with the molecular subtype of LUAD, we performed unsupervised clustering using 20 terms from the GO biological process and evaluated each major non-silent somatic mutation, including *MET*, *BRAF*, *EGFR*, *KEAP1*, *KRAS*, and *TP53*. We selected these six genes because of their high incidence of somatic mutations in LUAD. Three clusters were obtained and labeled 0, 1, and 2. The samples used for this analysis were primary tumor samples selected based on gene expression and clinical data. The initial sample count was 514. After selecting samples with mutation data, the final cluster consisted of 508 samples. Cluster 1 contained 146 samples, most of which did not have a *TP53* mutation, whereas cluster 2 contained 122 samples, most of which had a *TP53* mutation. Cluster 0 included 240 mixed samples (Figure 3A). 

A chi-square test comparing all clusters and *TP53* yielded a *p*-value of 2.2 × 10^−16^. Consequently, the *TP53* mutation status was significantly associated with *PLK4*-related pathways (Figure 3D). The prognosis for the three clusters differed significantly, with cluster 2, characterized by the highest *PLK4*-related pathway scores, being associated with poor prognosis in both OS and PFS (Figure 3B). The expression level of *PLK4* was relatively high in the group with *TP53* mutations (Figure 3C), and the ratio of *TP53* mutations correlated with the clusters divided by *PLK4*-related pathway scores (Figure 3D).

### 3.4. Identification of Potential Druggability in High PLK4-Related Pathways

**Figure 4:**
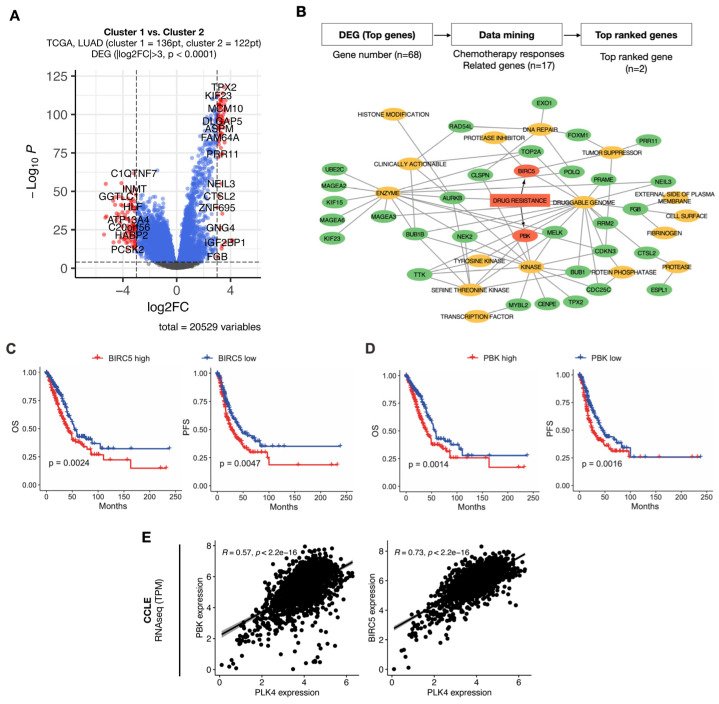
Comparison analysis between two clusters. Positive log2FC value indicates upregulation in cluster 2 (**A**). Network plot includes potential druggability terms from the drug–gene interaction (DGI) database and genes that were upregulated in cluster 2. Cutoff values for the genes used in plotting this network plot are log2FC > 3 and *p*-value < 0.0001 (**B**). Kaplan–Meier plots of *BIRC5* (**C**) and *PBK* (**D**) for OS and PFS. High- and low-expression groups were divided based on the median value of *BIRC5* and *PBK* expression. Results of linear regression correlations between *PLK4* and *PBK,* and *PLK4* and *BIRC5* are displayed (**E**).

We conducted a DEG analysis between clusters 1 and 2 to identify the potential druggability in the group with a high *PLK4*-related pathway signature associated with poor prognosis (Figure 4A). 

**Figure 5:**
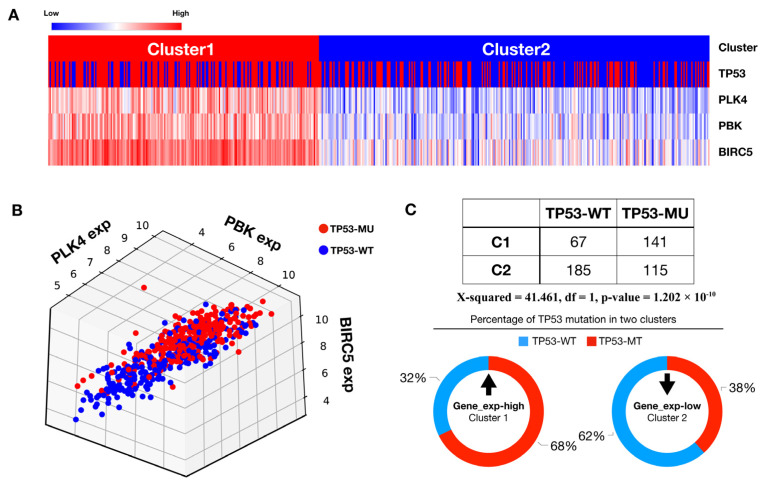
*TP53* mutation and *PLK4*-related gene expression can be an identification signature of LUAD. Two clusters were obtained through agglomerative clustering with the genes *PLK4*, *PBK*, and *BIRC5*, and a heatmap was plotted with *TP53* mutation information for each sample (**A**). On the cluster line, red corresponds to cluster 1 and blue corresponds to cluster 2. On the *TP53* line, red corresponds to *TP53* mutant (*TP53*-MU) and blue corresponds to *TP53* wild type (*TP53*-WT). A 3D scatter plot of gene expression for *PLK4*, *PBK*, and *BIRC5* is presented in coordinate space (**B**) (red dots represent *TP53* mutations and blue dots represent wild type). Results of a chi-square test between clusters and *TP53* mutations along with the percentage of *TP53* mutations in each cluster are visualized in a pie chart (**C**).

Sixteen druggability terms were represented using a drug–gene interaction database. The genes used to construct the network were selected by choosing genes with fold-change > 3 and *p*-value < 0.0001 from the DEG analysis obtained earlier, resulting in 68 genes. Among these 16 terms, we identified two genes, *BIRC5* and *PBK*, that were related to drug resistance (Figure 4B). Upregulation of these two genes was associated with poor prognosis in terms of both OS and PFS (Figure 4C,D). Additionally, the expression levels of *BIRC5* and *PBK* were highly correlated with *PLK4* expression across 1450 cell lines from the DepMap database (Figure 4E). Primary tumor samples with survival information were used for survival analysis, with a total of 505 samples. The analysis was stratified into high- and low-expression groups, with each group representing 50% of the total samples. Two previously identified genes, *PBK* and *BIRC5*, appear to be highly associated with *PLK4*. Two clusters were obtained through agglomerative clustering of *PLK4*, *PBK*, and *BIRC5* (Figure 5A) and were highly correlated with each of the three genes (Figure 5B). 

We confirmed that the clusters were significantly related to *TP53* mutations using a chi-square test (Figure 5C).

## 4. Discussion

Given that PLK4 significantly influences the cell cycle, numerous studies have investigated the antitumor effects of PLK4 inhibition [27,28]. The identification of genes that highly correlated with *PLK4* indicated that PLK4 did not function in isolation (Figure 2C). Most of these genes exhibited a high correlation with the cell cycle, further confirming the association of PLK4 with the cell cycle (Figure 2C). Through this occurrence, we confirmed that a higher *PLK4* expression was associated with a poor tumor prognosis and a high proportion of primary tumors (Figure 2A,B). Connecting this finding with bioinformatic data on *PLK4*, previous studies have reported a correlation between high *PLK4* expression and poor prognosis in various cancer types [24,25,29,30,31]. However, no connection has been established between LUAD and genes highly correlated with PLK4.

We identified three distinct clusters based on GO terms that exhibited strong correlations with *PLK4* expression (Figure 3A). After obtaining this cluster, we checked its correlation with six of the top-ranked genes (*TP53*, *KRAS*, *KEAP1*, *EGFR*, *BRAF*, and *MET*) among the various somatic mutations observed in LUAD and found that it was associated with a *TP53* mutation. When analyzing the correlation between each mutation and *PLK4* expression, we found that for *BRAF*, *KEAP1*, *MET*, and *KRAS* mutations, the association of *PLK4* expression was not significant, and for EGFR mutations, the association tended to decrease (Figure S1). However, *EGFR* mutations account for a small proportion of the total, and the role of PLK4 in TKI resistance in *EGFR*-mutated tumors has not yet been confirmed, so further studies are needed. The reason for these results is that the TCGA database typically comprises data obtained from surgical tissues, resulting in a limited diversity of somatic mutation samples. Among these clusters, cluster 2, which was characterized by a notable prevalence of *TP53* mutations in genomic variants, displayed elevated levels of *PLK4* (Figure 3C). This substantiates a significant association between PLK4 and *TP53* mutations, consistent with previous reports that highlighted a connection between *TP53* and PLK4 in cancer [32]. Our data do not demonstrate a relationship with other mutations, such as *EGFR* mutations and *ALK-* and ROS-rearranged mutations, which are highly prevalent in NSCLC. In the case of patients harboring these mutations, treatment typically involves the administration of tyrosine kinase inhibitors (TKIs). However, drug resistance is commonly observed in such cases. Investigating *PLK4* expression in TKI-resistant tumors and its role would be of interest. In future studies, we intend to gather data from patients treated with TKIs and analyze them, potentially yielding intriguing findings.

Building on the defined clusters, we conducted DEG analysis to identify genes that were prominently co-expressed with *PLK4*. We identified two specific genes, *BIRC5* and *PBK*, that are implicated in drug resistance (Figure 4B). The increased expression of these two genes was linked to an unfavorable patient prognosis (Figure 4C,D). Furthermore, these two genes were highly correlated with *PLK4* (Figure 4E), and we found a significant correlation of these genes with *TP53* mutations (Figure 5A,B). This compelling evidence underscores the potential of *PLK4* to serve as a pivotal therapeutic candidate to synergistically augment the efficacy of concurrent chemotherapeutic agents. This is particularly pertinent in the case of chemotherapy with drugs such as cisplatin, which plays a pivotal role in managing diverse lung cancer scenarios. Importantly, *BIRC5* and *PBK* have been previously reported to be associated with cisplatin resistance [33,34]. In a previous study, survivin, encoded by *BIRC5*, was reported to form a complex with caspase-9 and SMAC/DIABLO, preventing the mitochondrial pathway of apoptosis signaling, and studies on survivin as a target for overcoming cisplatin resistance have been conducted [33,35]. Similarly, previous in vitro and in vivo studies have shown that *PBK* induces autophagy through the ERK/mTOR signaling pathway, resulting in poor prognosis, metastasis, and cisplatin resistance [34]. These findings can guide the identification of diverse immunotherapeutic or chemotherapeutic combinations in the future, thereby bolstering the potential for comprehensive and efficacious treatment strategies. Recent clinical trials of CFI-400945 have mostly centered on add-on treatment of solid tumors with chemotherapy. (NCT01954316, NCT03624543). However, we have recently started to apply PLK4 inhibitors as combination therapy with durvalumab, a targeted therapy against PD-L1, in some solid tumors (NCT04176848), and we expect that PD-L1 and PD-1 are not unrelated to high outcomes in *PLK4* (Figure S2). This shows promise for combination therapy with immune checkpoint inhibitors targeting the PD-(L)1 axis.

## 5. Conclusions

In summary, patients with LUAD with *TP53* mutations exhibit a high *PLK4* expression, which is also associated with poor prognosis. Our study introduces PLK4 as a therapeutic target and prognostic biomarker in patients with LUAD. This suggests that signature genes related to *PLK4* could serve as predictive biomarkers for the development of therapeutic agents targeting PLK4. Building on previous investigations that have highlighted the correlation between *TP53* mutations and the tumor immune microenvironment [36,37,38], an in-depth exploration of the interplay between PLK4 and immunity is imperative. Using a cellular assay and syngeneic and NOD/SCID mouse model, a previous study showed that the mutant p53 protein blocks the formation of the STING-TBK1-IRF3 trimeric complex that inactivates innate immune signals, leading to immune evasion [38]. The repertoire of genes linked to drug resistance identified in a complementary manner holds promise for enhanced antitumor effects through synergistic combination strategies with PLK4 inhibitors in prospective therapeutic landscapes. This combined approach could potentially lead to an increased efficacy against malignancies. Our study significantly contributes to understanding LUAD by identifying a substantial link between *TP53* mutations and increased *PLK4* expression. These observations highlight the usefulness of PLK4 not only as a potential therapeutic target but also as a prognostic biomarker. The potential use of *PLK4*-related signature genes as predictive biomarkers for PLK4-targeted therapies represents a novel approach to individualized treatment strategies. We also conducted correlation analyses between *PLK4* and several genes associated with cell invasion and proliferation. Our findings revealed a significant positive correlation with genes related to proliferation, as well as a notable but moderately positive correlation with genes linked to cell invasion (Figure S3). Furthermore, among the genes associated with cell invasion, *TWIST1* is also known to have implications for TGF-beta-related signaling, which suppresses the immune system. Hence, we anticipate that future research will shed light on the connection between PLK4 and the immune system, offering potential insights into the immunotherapy targeting PLK4 (Figure S3B). However, the mechanisms underlying the observed association between *TP53* mutations, *PLK4* expression, and poor prognosis have not yet been fully elucidated. Further experimental work, such as functional studies and molecular investigations, will be needed to elucidate the molecular pathways linking these elements.

## Data Availability

The data presented in this study are available in this article.

## Supplementary Materials

The following supporting information can be downloaded at https://www.mdpi.com/article/10.3390/cancers15184663/s1, Table S1: A total of 87 *PLK4*-related genes through Pearson’s correlation analysis with correlation coefficient above 0.8. Table S2: A total of 87 *PLK4*-related genes significantly enriched in GO biological process terms. Figure S1: Correlation analysis result of *PLK4* expression with *BRAF*, *EGFR*, *KEAP1*, *MET*, and *KRAS* mutations. Figure S2: Results of *PLK4* expression analysis in LUAD patient samples based on differences in expression of PD-1 and PD-L1 (threshold: 50%). Figure S3: Correlation between *PLK4* and genes related to proliferation and invasion.
