# Supplementary material for: Polo-like Kinase 4: A Multifaceted Marker Linking Tumor Aggressiveness and Unfavorable Prognosis, and Insights into Therapeutic Strategies
Source: Cancers (Basel). 2023 Sep 21;15(18):4663. doi: 10.3390/cancers15184663 (PMC10526937; doi:10.3390/cancers15184663)
Supplement: Supplementary file 1 [file cancers-15-04663-s001.zip › Certification.pdf]

Editing Certificate

This document certifies that the manuscript listed below has been edited to ensure language and grammar accuracy and is error free in these aspects. The logical presentation of ideas and the structure of the paper were also checked during the editing process. The edit was performed by professional editors at Editage, a brand of Cactus Communications. The author's core research ideas were not altered in any way during the editing process. The quality of the edit has been guaranteed, with the assumption that our suggested changes have been accepted and the text has not been further altered without the knowledge of our editors.

MANUSCRIPT TITLE

PLK4 : A Multifaceted Marker Linking Tumor Aggressiveness and Unfavorable Prognosis, and Insights into Therapeutic Strategies

AUTHORS

Youngtaek Kim, Joon Yeon Hwang, Dong Kwon Kim, Kwangmin Na, Seul Lee, Sujeong Baek, Seong-san Kang, Seung Min Yang, Mi Hyun Kim, Heekyung Han, Chai Young Lee, Yu Jin Han, Min Hee Hong, Jii Bum Lee, Byoung Chul Cho, YoungJoon Park, Kyoung-Ho Pyo

ISSUED ON

August 19, 2023

JOB CODE

GHEKK_1

THE EDITAGE Promise QUALITY GUARANTEE

Prabh Grewal
Vice President, Operations - Editage

editage helping you get published

Since 2002, Editage has helped over 430,000 authors publish around 1.2 million research papers in scholarly journals across over 1000 disciplines through editorial, translation, transcription, and publication support services. Editage is a brand of Cactus Communications (cactusglobal.com), a science communication and technology company.

CACTUS

GLOBAL :
+1(833) 979-0061 | request@editage.com

KOREA :
02-3478-4396 | submit-korea@editage.com
